# Personality Counts More Than Appearance for Men Making Affective Judgments of Verbal Comments

**DOI:** 10.3389/fpsyg.2019.00078

**Published:** 2019-01-25

**Authors:** Shan Gao, Lizhu Luo, Wanyu Zhang, Yuxin Lan, Ting Gou, Xun Li

**Affiliations:** ^1^School of Foreign Languages, University of Electronic Science and Technology of China, Chengdu, China; ^2^The Clinical Hospital of Chengdu Brain Science Institute, MOE Key Laboratory for NeuroInformation, University of Electronic Science and Technology of China, Chengdu, China

**Keywords:** praise, criticism, personality, appearance, gender

## Abstract

Previous research has shown that that evaluative verbal information (praise and criticism) conveys different affective values: criticism is perceived as unpleasant while praise is generally considered pleasant. Here, using praise and criticism in Chinese, we investigated how affective value is modulated in men and women, depending on the particular attribute (personality vs. appearance) targeted by social comments. Results showed that whereas praise was rated as pleasant and criticism as unpleasant overall, criticizing personality reduced pleasantness more than criticizing appearance. In men, moreover, criticism of personality was deemed more unpleasant than criticism of appearance while personality-targeted praise was rated more pleasant than appearance-targeted praise. This effect was absent in women and consistent with men’s higher arousal ratings for personality- relative to appearance-targeted comments. Our findings suggest that men are more concerned about external perception of their personality than that of their appearance whereas women’s affective judgment is more balanced. These gender-specific results may have implications for topic selection in evaluative social communication.

## Introduction

Humans make comments on each other in daily interactions. Positive comments such as praise and appreciation induce happiness and pride; in contrast, negative comments including personal criticism, disapproval and rejection make recipients angry and sad (Carleton et al., 2006). Previous studies have shown that differentially valenced comments, praise or criticism, trigger different emotional responses: criticism is perceived as unpleasant whereas praise pleasant in both healthy subjects (Blair et al., 2008; Miedl et al., 2016) and patients with generalized social phobia (Blair et al., 2008).

However, little is known about how emotional responses to criticism and praise change as a function of respects on which these comments are made. Our prior work (Gao et al., 2017) examined whether females prefer self-referential metaphoric/literal compliments paid by males targeting their appearance or possessions. We found that paired with compliments on appearance as compared to possessions the attraction of neutral faces of male individuals was increased, indicating the impact of compliment target in a mate-selection context. Here, we engaged different targets of criticism and praise, appearance and personality, and investigated their modulation of affective judgments in a generic sense of social evaluation. Evidence in literature demonstrates that appearance and personality may weigh differently in making a variety of decisions. In personnel-selection, physical appearance counts more in high self-monitoring individuals while in low self-monitoring individuals job-appropriate personality does more (Snyder et al., 1988). In evaluation of political candidates, physical appearance has robust effects even when individuating personality information is provided (Budesheim and DePaola, 1994). Therefore, we expected that affective judgments would differ when self-referential criticism and praise are posed on appearance and personality.

More importantly, we hypothesized these appearance-, personality-induced differences would be modulated by gender. Men and women have different gender roles, which determine how they think and act in a society (Lenton, 2010). They have been stereotyped as “breadmakers” and “housekeepers,” respectively although these prejudices may be more prevailing in certain cultures than in others (Ma et al., 2008). For men, who are traditionally associated with social production, work-related characteristics like personality and competence may be more valuable than physical appearance. Evidence has shown that men regard all attributes of appearance except height as less important than women do (Jackson et al., 1987). Therefore, we hypothesized that personality values more in men rather than in women so that in men commenting on personality relative to appearance would increase the pleasantness of praise and unpleasantness of criticism.

## Methods

### Participants

Sixty healthy Chinese volunteers (30 males, mean age 21.75 years, ranging from 18 to 30) were recruited by local advertisement. All participants gave written informed consent in accordance with the Declaration of Helsinki. The protocol was approved by the local ethics committee at the University of Electronic Science and Technology of China.

### Stimuli

The experiment materials were praising and criticizing sentences of 3 to 7 Chinese characters (*M* = 4.64, *SD* = 1.003). They focused on gender-independent attributes of personality and appearance, encompassing comments of four categories (50 of each): appearance-targeted praise (e.g., “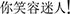,” “Your smile is charming!” in English translation), appearance-targeted criticism (e.g., “
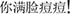
” “Your face is spotty!” in English), personality-targeted praise (e.g., “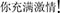,” “You are passionate!” in English) and personality-targeted criticism (e.g., “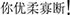,” “You are indecisive!” in English). Appearance-targeted comments were based on various attributes of face and physiques such as eyes, nose, lips, hairs, skin, waistline, hips, legs, body height and weight.

### Procedure

All sentences were presented randomly using E-prime 2.0 software and none of them was repeated. Participants read every sentence as a description about themselves given by an acquaintance and then rated, using 7-point scales, how much they were emotionally aroused and pleased by the comments. In each trial, preceded by a fixation-cross for a random duration between 300 and 700 ms, a sentence was displayed for 1000 ms and then followed by cues for rating arousal and valence consecutively. The duration of each rating was time-locked to the key press and the next trial was initiated after participants finished both ratings.

### Data Analysis

Using IBM SPSS Statistics version 22 ratings were analyzed employing three-way repeated-measures ANOVAs with comment-type (praise vs. criticism) × comment-target (appearance vs. personality) as within-subjects variables and gender as a between-subjects variable. Partial eta squared ($\eta_{p}^{2}$) was calculated as a measure of effect size. Bonferroni correlation was used when pairwise comparisons were applicable.

## Results

### Pleasantness

**FIGURE 1:**
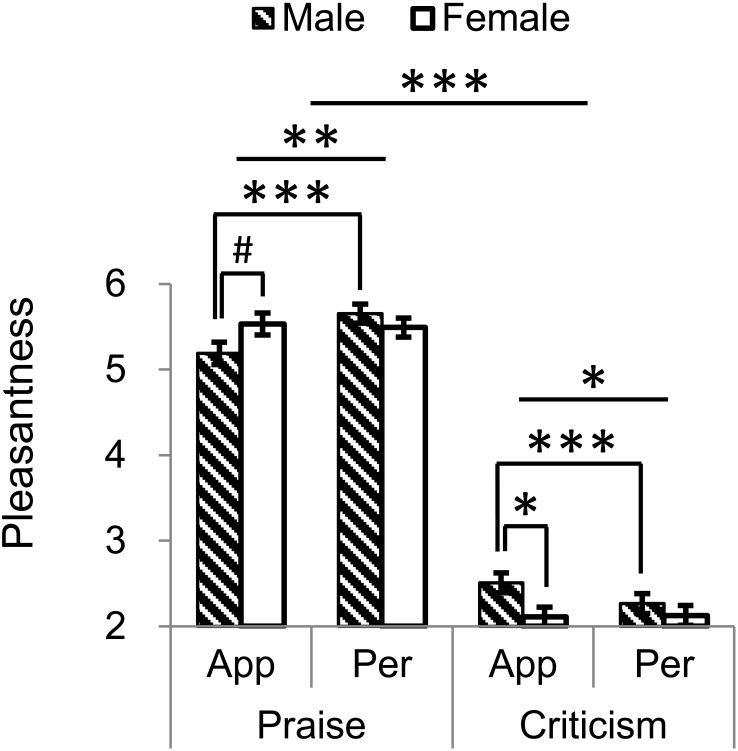
Valence ratings for praise and criticism targeting appearance and personality in males and females. There were a comment-type main effect, type × target interaction and type × target × gender interaction. ^∗^*P* < 0.05; ^∗∗^*P* < 0.01; ^∗∗∗^*P* < 0.001; ^#^*P* = 0.064. Bars depict M ± SE. App, appearance; Per, personality.

In terms of pleasantness (Figure 1), we observed a significant main effect of comment-type (*F*_1,58_ = 674.622, *P* < 0.001, $\eta_{p}^{2}$ = 0.921) with praise rated as pleasant (*M* ± SE = 5.467 ± 0.076) and criticism unpleasant (2.252 ± 0.079). There was an interaction between comment-type and comment-target (*F*_1,58_ = 12.76, *P* = 0.001, $\eta_{p}^{2}$ = 0.18). Pairwise comparisons revealed that targeting personality relative to appearance praise became even more pleasant (*M*_personality_ -*M*_appearance_ = 0.211 ± 0.074, *F*_1,58_ = 8.119, *P* = 0.006, $\eta_{p}^{2}$ = 0.123) and criticism even more unpleasant (*M*_personality_ -*M*_appearance_ = -0.115 ± 0.044, *F*_1,58_ = 6.697, *P* = 0.012, $\eta_{p}^{2}$ = 0.104).

We also found a three-way interaction of gender with comment-type and comment-target (*F*_1,58_ = 17.772, *P* < 0.001, $\eta_{p}^{2}$ = 0.235). Whereas both sexes rated praise as more pleasant than criticism regardless of target, in males praise on personality as compared to appearance was even more pleasant (*M*_personality_ -*M*_appearance_ = 0.465 ± 0.105, *F*_1,58_ = 19.688, *P* < 0.001, $\eta_{p}^{2}$ = 0.253) while criticism on personality relative to appearance was even more unpleasant (*M*_personality_ -*M*_appearance_ = -0.245 ± 0.063, *F*_1,58_ = 15.328, *P* < 0.001, $\eta_{p}^{2}$ = 0.209). These effects were not observed in females (*P*s > 0.6). On the other hand, males perceived appearance-based criticism less unpleasant than females did (*M*_male_ -*M*_female_ = 0. 399 ± 0.163, *F*_1,58_ = 6.002, *P* = 0.017, $\eta_{p}^{2}$ = 0.094) while a marginally significant reduction of pleasantness was found in males as compared to females for appearance-targeted praise (*M*_male_ -*M*_female_ = -0. 345 ± 0.183, *F*_1,58_ = 3.558, *P* = 0.064, $\eta_{p}^{2}$ = 0.058). No other main or interactive effects were yielded (*P*s > 0.1).

### Arousal

**FIGURE 2:**
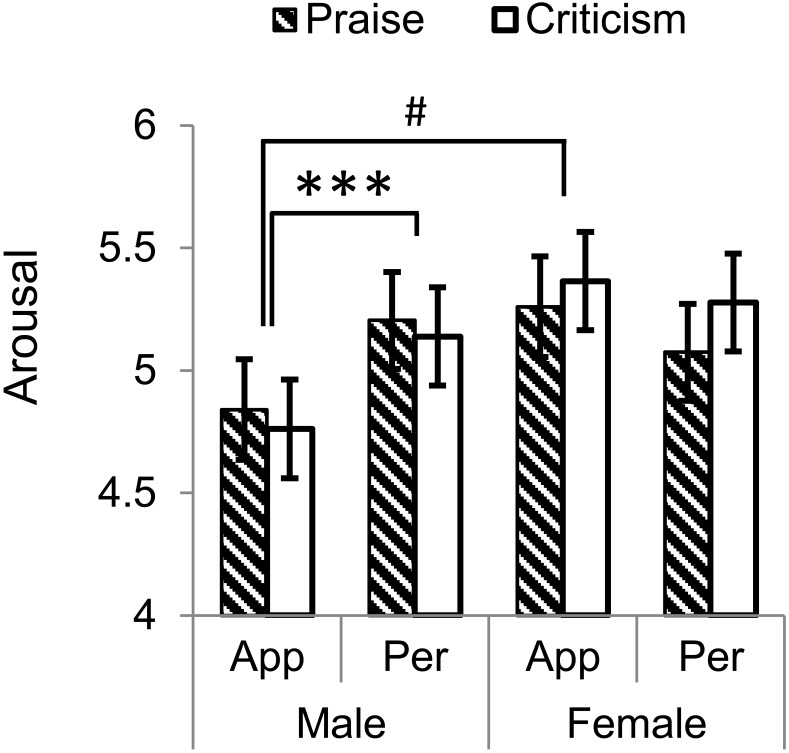
Arousal ratings for praise and criticism targeting appearance and personality in males and females. An interaction between comment-target and gender was found showing that males were more aroused by personality-targeted comments than appearance-targeted ones. ^∗∗∗^*P* < 0.001; ^#^*P* = 0.064. Bars depict *M* ± SE. App, appearance; Per, personality.

Arousal ratings (Figure 2) displayed a significant interaction between comment-target and gender (*F*_1,58_ = 12.9, *P* = 0.001, $\eta_{p}^{2}$ = 0.182). In males comments on personality were more arousing than those on appearance (*M*_personality_ -*M*_appearance_ = 0.371 ± 0.1, *F*_1,58_ = 13.772, *P* < 0.001, $\eta_{p}^{2}$ = 0. 192), while females were not aroused differentially by appearance- or personality-directed comments (*M*_personality_ -*M*_appearance_ = -0.137 ± 0.1, *F*_1,58_ = 1.872, *P* = 0.176, $\eta_{p}^{2}$ = 0.031). There was a trend of gender difference in ratings for appearance-targeted comments with men being less aroused than women (*M*_male_ -*M*_female_ = -0.512 ± 0.217, *F*_1,58_ = 3.571, *P* = 0.064, $\eta_{p}^{2}$ = 0.058). No main or any other interactive effects were identified (*P*s > 0.1).

## Discussion

This study measured the differences in emotional reactions indexed by subjective evaluation when people are receiving praising and criticizing comments on their own physical appearance and personality traits. The results supported our hypothesis, showing a modulatory effect of different targets of verbal comments on emotional responses and its gender-specificity.

Overall, praise was perceived as pleasant and criticism unpleasant, which is consistent with previous findings (Carleton et al., 2006; Blair et al., 2008; Miedl et al., 2016). Importantly, this valence effect was altered by different comment targets with increased pleasantness for praise and unpleasantness for criticism of personality relative to appearance. Note that all these effects were not confounded by emotional intensity across different comment types and targets where there were no variations in arousal. Our previous work (Gao et al., 2017) observed, in a romantic scenario, a regulation of men’s attractiveness to women when they make compliments on women’s appearance and possessions. The present study further shed light on the essential role of attributes on which evaluative comments are made in more generic social interaction.

Intriguingly, our manipulations of verbal comments interplayed with gender. Males were more pleased by praise on their personality relative to appearance while they were unhappier with being criticized of their personality as compared to appearance. Differentially, females showed no such effect of target. This indicates that men, not women, are likely to weight affectively their personality more than their appearance in social evaluation and it is further supported by higher arousal of personality-targeted comments in men. On the other hand, receiving appearance-targeted criticism men, as compared to women, displayed attenuated unpleasantness. Similarly, pleasure was reduced for praise of appearance in men relative to women. Men seemed to be less aroused by appearance-oriented comments than women. This is in line with previous finding that men consider all appearance attributes except height as less important than women do (Jackson et al., 1987). Taken together, our findings provide the first evidence for gender-dependent modulation of emotional responses by verbal comments on different attributes of a person.

Gender differences observed here may be contributed by distinct gender roles that have long been influencing attitudes and behaviors in men and women. Men are traditionally presumed to be leaders, heads of their households by providing financial support for the family and making important family decisions. Therefore, personal traits and abilities rather than physical appearance are highly valued in men, at least in male dominant societies (Cuddy et al., 2015). Enhanced emotional reactions in men here to comments on personality and competence rather than appearance indeed fit into their masculine gender stereotype with strength and control. Differently, women have been subordinate with less power and this gender bias has not been eliminated (Zhang, 2006; McKay, 2013). Women tend to please men and satisfy societal expectations by taking their domestic role and keeping themselves physically attractive. In traditional Chinese culture, physical attraction of a woman not only gives pleasure to a man but also earns credits for his social status. More generally, both sociocultural and personal evaluation standards for feminine identity place greater emphasis on physical appearance than do those for men, which even occurs to well-educated women (Cash et al., 1997). However, our findings suggest that women, praised of personality, are as happy as when compliments are addressed on their appearance. And they are upset to a similar extent by criticism of both categories of attributes. This may indicate a positive consequence of changing gender role attitude in women. Despite a decline in gender inequality over time (He and Wu, 2017), it nevertheless seems that appearance still weighs more for women than for men, leading to more pleasure in women than in men when praised of appearance. This finding is consistent with previous research suggesting that females continually seek reassurance of their appearance to facilitate social acceptance (McKay, 2013).

It is noteworthy that language use and gender roles are influenced by particular cultures, which have, however, been interacting with each other across regions and nations (Lin et al., 2012; Zuo et al., 2018). In the present study, gender-specific modulation of emotional reactions to differently oriented evaluations may be contributed by cultural preferences in China. Future work can examine whether these effects are generalizable to other cultures. Another limitation is that this study did not specify the sex of comment-givers, which may interplay with that of comment-receivers in evaluative discourse (Herbert, 2008). It is important to investigate in future how differently men and women react to homosexual and heterosexual evaluations and thus provide suggestions for facilitating sex-related social communications and relations.

In conclusion, the present study demonstrated, from a perspective of affective function, gender-dependent modulation of different topics in evaluative language, which may be contributed by gendered values of appearance and personality attributes in Chinese culture. As traditional “breadmakers,” men emotionally concern more about personality relative to appearance. While women show similar reaction to personality- and appearance-oriented evaluations they feel happier than men when praised of appearance. Overall, these findings suggest that appropriately gendered selection of topics in evaluation-based discourse may facilitate social interactions. To compliment men, personality is a better choice than appearance whereas caution should be exercised in criticizing it.

## Data Availability

The raw data supporting the conclusions of this manuscript will be made available by the authors, without undue reservation, to any qualified researcher.

## Author Contributions

SG and LL conceived the study. SG, WZ, YL, TG, and XL collected and analyzed the data. SG, LL, WZ, YL, and TG interpreted the results and wrote the manuscript. All authors discussed the results and commented on the manuscript.

## Conflict of Interest Statement

The authors declare that the research was conducted in the absence of any commercial or financial relationships that could be construed as a potential conflict of interest.
